# Development loop-mediated isothermal amplification assay for detection of enteropathogenic *Escherichia coli* (EPEC)

**DOI:** 10.1007/s11033-026-12206-x

**Published:** 2026-06-26

**Authors:** Alazar Amare Amdiyee, Tesfaye Sisay Tessema

**Affiliations:** 1 Biotechnology Research Center, Addis Ababa University, Addis Ababa, Ethiopia; 2 College of Medicine and Health Science, Department of Medical Laboratory Sciences, Dilla University, Dilla, Ethiopia

**Keywords:** EPEC, LAMP, PCR, Sensitivity, Specificity, Molecular diagnostic

## Abstract

**Background:**

Enteropathogenic Escherichia coli (EPEC) is a major etiological agent of persistent diarrhea in pediatric and adult population globally and it is a leading cause of infant mortality in developing countries. Bacterial culture and biochemical assays are insufficient for accurately identifying EPEC strains. In contrast, PCR serves as a major molecular diagnostic method for EPEC. However, the application of PCR is limited due to high cost, time taking and technical requirements.

**Method and Results:**

In this study loop mediated isothermal amplification LAMP) was developed for rapid, specific, easy and resource-friendly detection of EPEC. The LAMP assay was developed by designing specific lamp primers targeting eae genes, in combination with stx1 and stx2 lamp primers to enable EPEC detection. The reaction mixture, which contained 2.5 µL of isothermal amplification buffer, 10 mM of MgSO4 solution, 1.4 mM of dNTPs, 1.8 mM of FIP and BIP, 0.4 mM of F3 and B3, 0.2 mM of LB, 8U of Bst polymerase, and 2.0 µL of the target DNA template, was incubated at 62 C0 for 60 minutes. The designed lamp assay's performance was assessed utilizing 60 bacterial trains that were isolated locally. The assay attained 100% efficiency (60/60), 100% sensitivity (10/10), and 100% specificity (50/50). Furthermore, both the positive and negative predictive scores were 100%. Up to 0.05 pg of DNA could be detected in each reaction using the developed LAMP test. The traditional PCR, on the other hand, showed a detection limit of 5 pg/reaction. Additionally, the developed LAMP assay was able to detect up to 7 x 102 cfu/g stool in a spiked stool sample. PCR, however, can identify up to 7 x 104 cfu/g of stool. Notably, compared to conventional PCR, the LAMP assay has a 100-fold greater sensitivity. The result obtained from LAMP and PCR tests showed a perfect agreement with a kappa value of 1 (k = 1).

**Conclusion:**

The developed LAMP assay demonstrated promising performance for the detection of EPEC under controlled laboratory conditions, offering a rapid and straightforward alternative to conventional diagnostic methods. Clinical validation using patient stool sample and further studies with larger sample sets are needed to fully establish the assay's performance and support its broader clinical application.

**Supplementary Information:**

The online version contains supplementary material available at 10.1007/s11033-026-12206-x.

## Introduction

One of the main causes of diarrheal disease worldwide is diarrheagenic *Escherichia coli* (DEC), a varied group of pathogenic E. coli. In low- and middle-income countries (LMICs), DEC infections dramatically raise morbidity and death, particularly in young children and infants [1]. They are divided into separate pathogenic groups as: Enteropathogenic *Escherichia coli* (EPEC), Enterohemorrhagic *Escherichia coli* (EHEC), Enterotoxigenic *Escherichia coli* (ETEC), Enteroinvasive *Escherichia coli* (EIEC), Enteroaggregative *Escherichia coli* (EAEC), Shiga toxin-producing *Escherichia coli* (STEC), Diffusely adherent *Escherichia coli* (DAEC) and Cell-detaching *Escherichia coli* (CDEC) strains based on their preferred virulence mechanisms, host colonization sites, and clinical symptoms [1–3]. Enteropathogenic *Escherichia coli* (EPEC) is one of the major DEC strains that causes chronic diarrhea in adults and children worldwide. It is still a major contributor to childhood morbidity and mortality, especially in developing countries, and it is connected to outbreaks in both community and medical settings [4, 5].

EPEC strains induce attaching and effacing (A/E) lesions on intestinal epithelial cells, mediated by the intimin protein encoded by eae gene. All EPEC strains that are intimin (eae) +, bundle forming protein (bfpA) + and shiga toxin genes (stx)*−* classified as typical EPEC strains, whereas atypical strains are intimin (eae) +, bundle forming protein (bfpA) − and *−* for shiga toxin (stx) genes. Consequently, all EPEC strains, whether typical or atypical, are stx − and eae+, reflecting their distinct pathogenic profile [6, 7]. Bacterial culture and biochemical assays are insufficient to detect EPEC strains since they cannot be distinguished from normal flora and other pathogenic E. coli strains. However, polymerase chain reaction (PCR) is an important molecular technique for detecting EPEC strains based on the presence or absence of specific virulence genes [8]. However, because PCR requires expensive equipment, time, skilled personnel, and complex laboratory setup, it is less accessible for frequent usage in environments with limited resources. However, the invention of loop-mediated isothermal amplification (LAMP) offers a workable alternative by making target pathogen identification simple, rapid, and inexpensive [9, 10].

Notomi et al. first described LAMP, a new nucleic acid amplification method, in 2000. In contrast to PCR, LAMP uses a large segment of Bacillus stearothermophilus (Bst) DNA polymerase for auto-cycling strand displacement DNA synthesis while operating in an isothermal environment. This technique increases the assay’s sensitivity and specificity by using a series of four to six specially created primers that identify eight different sections of the target sequence. LAMP is a very effective and dependable molecular diagnostics tool because of its quick amplification of the target nucleic acids under isothermal conditions [11–13]. Additionally, the LAMP assay was carried out using quite basic and reasonably priced equipment, such as a water bath and a heat block that can maintain a steady temperature, making them simple to use in regular laboratories [14]. Mycobacterium tuberculosis, measles virus, malaria parasites, human papillomavirus, Vibrio cholerae, and COVID-19 are just a few of the pathogens that have recently been successfully detected using loop-mediated isothermal amplification (LAMP) [15–21]. The objective of this study was to developed loop mediated isothermal amplification assay which is rapid, sensitive, specific, resource-friendly and easy method for EPEC detection.

## Materials and methods

### Bacterial strains

60 bacterial strains 10 EPEC, 10 ETEC, 10 STEC, 10 EAEC, 2 EIEC, 4 EHEC, 10 non-pathogenic E. coli strains, and 4 non-*E. coli* bacterial species (*Staphylococcus aureus*,* Pseudomonas aeruginosa*,* Salmonella typhi*,* and Klebsiella pneumoniae*) that were available in glycerol stocks at Addis Ababa University, Biotechnology Research Center were used to develop, optimize, and evaluate the LAMP assay. Eosin methylene blue (EMB) agar was used to cultivate *E. coli* strains, while nutrient agar was used to cultivate non-*E. coli* bacteria for 24 h at 37 °C. The isolates were then subcultured in 10 ml of TSB for 24 h at 37 °C.

### DNA extraction

A 1.5 ml Eppendorf tube was filled with one ml of each bacterial culture in tryptone soya broth (TSB), and the tube was centrifuged for ten minutes at 15,000 rpm. After discarding the supernatants, the pellet was resuspended with 200 µL of nuclease-free water and centrifuged for 10 min at 15,000 rpm. The pellet was resuspended with 100 µL of TE buffer and incubated at 100 C^0^ for 10 min after the supernatant was discarded. After centrifuging it for one minute at 10,000 rpm and cooling it on ice, the supernatant was moved to a fresh, sterile Eppendorf tube and kept at -20 °C until it was needed as the DNA template for PCR and LAMP. The concentration and purity of the extracted DNA were measured using the Nanodrop 2000/2000 C Spectrophotometer (Thermo ScientificTM, USA).

### PCR amplification

The conventional PCR assay was performed in parallel to validate the result of LAMP assay using specific PCR primers (Table 1). Each PCR assay was performed in 25 µl final reaction volume containing, 2.5 Mm of PCR buffer, 2.5 Mm of MgCl_2_, 0.35 millimolar (mM) of each dNTP, 0.5 µM of each forward and reverse primers, 0.5 µl of Taq DNA polymerase (5U/µl, Solis BioDyne),14 µL of nuclease free water and 3 µL of DNA template. The same reaction mixture without template DNA was used as a negative control. Amplification was carried out with an initial denaturation temperature of 94 °C for 5 min followed by 35 cycles of each consisting of 40 s of denaturation, 40 s of annealing ,1 min of extension. The last amplification cycle included an additional step of final extension at 72 °C for 10 min. All amplifications were carried out in a Prima 96 plus thermal cycler (Himedia India). The PCR product was subjected to 1.5% agarose gel electrophoresis to analyze DNA fragments. The electrophoresis was carried out at 100 V for 1 h in 1X TAE buffer. A 100-bp DNA molecular weight marker was used to estimate the product size. The resulting DNA bands were visualized and documented using a UV gel documentation system.

**Table 1:** Nucleotide sequences and amplification characteristics of the PCR primers

Target	Primer name	sequence 5` − 3`	Anneling temprature.	Product length	Ref.
eae	F	AAACAGGTGAAACTGTTGCC	56 °C	450	[22]
	R	CTCTGCAGATTAACCTCTGC			
Stx 1	F	ATCAGTCGTCACTCACTGGT	55 °C	110	[23]
	R	CTGCTGTCACAGTGACAAA			
Stx 2	F	CAACACTGGATGATCTCAGC	55 °C	350	[23]
	R	CCCCCTCAACTGCTAATA			

### LAMP assay and detection of amplification products

The LAMP assay reaction setup including: a 25 µL reaction mixture containing 1 µL of Bst DNA polymerase (8 U, New England Biolabs), 2.5 µL of Isothermal Amplification Buffer, 10.0 mM MgSO₄, 1.2 mM dNTPs, 1.6 µM each of FIP and BIP primers, 0.2 µM each of F3 and B3 primers, 0.4 µM each of LF and LB primers, and 2.0 µL of template DNA. Sterile double-deionized water (ddH₂O) was used as a negative control in place of template DNA. The mixture was incubated at 65 °C for 60 min and terminated by heating at 80 °C for 5 min.

In this study the presence of LAMP amplification product was confirmed by using a combination of detection methods, including visual turbidity assessment, SYBR Green I fluorescence staining, and agarose gel electrophoresis. SYBR Green I detection was performed by adding 1µL of SYBR Green I to the end product, and the color in the mixture was visualized by the naked eye SYBR green has the ability to bind the minor groove of the dsDNA, whenever there are amplification products the intensity of the fluorescent will increase as the result of color change from orange to bright green. In the Prescence of amplified DNA products the SYBR green turned to green color, while the reaction without DNA amplification remained orange SYBR gold dye. For the gel electrophoresis analysis, 3 µL of each reaction product was subjected to 1.5% agarose gel stained with ethidium bromide; then, electrophoresis was carried out by 100 V for 1 h in 1X TAE buffer. In the presence of target amplification, a ladder of DNA bands was visualized and documented by using UV transilluminator gel documentation system. The amplification product was also confirmed by using visual turbidity assessment.

### Determination of developed lamp assay’s sensitivity, specificity, and detection limit

By using 60 bacterial isolates including 10 enteropathogenic *Escherichia coli* (EPEC) strains and 50 non-EPEC bacterial isolates the developed LAMP assay’s performance was assessed. The availability of samples limits the number of isolates that can be used in the present study. However, the strain contains a range of target and non-target isolates, enabling the assessment of the developed LAMP assay’s analytical sensitivity and specificity. Sensitivity was defined as the proportion of EPEC strains correctly identified as positive, while specificity referred to the proportion of non-EPEC strains accurately classified as negative. These criteria were used to assess the assay’s capability in detecting EPEC under defined experimental conditions. For determining lower detection limit of the developed LAMP assay, overnight broth cultures of *E. coli* strain was serially diluted from 10^− 1^ up to 10^− 10^. Briefly, a single colony of each strain was inoculated separately into 10 ml of fresh TSB and incubated at 37 °C for 24 h. The cultures were 10-fold serially diluted in normal saline, then an aliquot of 1 ml of each dilution was used for DNA template preparation and another 1 ml plated on plate count agar for CFU count to evaluate the detection limit of the assay in terms of lowest numbers of cells that could be detected. For determining lower number of DNA concentration/reaction the extracted DNA from original culture was subjected to 10-fold serial dilutions ranging from 10⁻¹ to 10⁻¹⁰. Two µL of template DNA from each dilution was used for LAMP and PCR assay. Finally, the lower detection limit of the assay was presented as in terms of lower number of DNA concentration/reaction and numbers of CFU/ml.

### Application of LAMP to EPEC-spiked human fecal samples

10 separate human fecal samples, each weighing one gram, were collected from a healthy donor and diluted in nine milliliters of phosphate-buffered saline (PBS). After vortexing the samples for ten seconds, letting them settle for two minutes, and then adding one ml of the stool suspension to ten different tubes, one ml of serially diluted (10^0^ to 10^10^) EPEC bacterial suspension was added. A 10X EDTA-filled Eppendorf tube was then filled with 1 ml of each spiked fecal sample dilution. After 30 s of vortexing, the samples were centrifuged for 15 min at 15,000 rpm. After discarding the supernatants, 300 µl of nuclease-free water was added, and the centrifugation process was repeated for 10 minutes at 15,000 rpm. The pellets were suspended in 100 µl of 1x TE buffer and boiled at 1000 C for 15 min after the supernatants were discarded. Centrifugation was carried out at 15,000 rpm for five minutes following boiling. After that, the supernatant was moved to a fresh, sterile Eppendorf tube to be utilized as template DNA. The template was then put through the PCR and LAMP experiment. The negative control was the feces devoid of EPEC.

### Data analysis

A 2 × 2 contingency table was used to assess the diagnostic performance metrics including sensitivity, specificity, positive predictive value (PPV), negative predictive value (NPV), and accuracy and evaluated with MedCalc software version 23.11.3. Furthermore, the degree of agreement between the PCR and LAMP assay results was assessed using Cohen’s kappa (κ) statistic.

## Results

### Primer design for LAMP assay

In this study a set of 6 primers were designed by targeting 5’ conserved region of attaching and effacing (eae) gene (Gene Bank accession no: M58154) [24].The software available at the following webpages; https://www.optigene.co.uk/custom-services/lamp-designer software, http://lamp.neb.com was used to design the primer. These include outer primers (Forward outer primer-F3 and Backward outer primer-B3), inner primers (Forward inner primer-FIP and Backward inner primer-BIP) and Loop primers (Forward loop primer-FLP and Backward loop primer-BLP). The designed primer set was used in conjunction with previously reported LAMP primers for stx1 and stx2 genes to confirm the absence of Shiga toxin genes to rule out STEC/EHEC, ensuring specific identification of EPEC strains. The overall information of primer sequences for eae, stx1 and stx2 are provided in Table 2.

**Table 2:** Designed LAMP primer sequences and their corresponding target gene for EPEC detection

Target	Name	Sequence 5` − 3`	Length (bp)	Ref.
eae	FIP(F1C-F2)	AGCTGGCTACCGAGACTCGC-AAGCAACATGACCGATGACA	40	This study
	BIP(B1C-B2)	CGCGATCTCTGAACGGCGATCCTGCAACTGTGACGAAGC	39	
	F3	AATGTCCCCGGACGTGAC	18	
	B3	GATTAACCTCTGCCGTTCCA	20	
	FLP	GTTGTGCCGCATAATTTAATGCCT	24	
	BLP	AAAGATACCGCTCTTGGTATCGCT	24	
Stx1	FIP	CCTGCAACACGCTGTAACGTCAGGTACAACAGCGGTTA	38	[25]
	BIP	AGTCGTACGGGGATGCAGATAGTGAGGTTCCACTATGC	38	
	F3	TGATTTTTCACATGTTACCTTTC	23	
	B3	TAACATCGCTCTTGCCAC	18	
	FL	GTATAGCTACTGTCACCAGACAATG	25	
	BL	AAATCGCCATTCGTTGACTACTTCT	25	
Stx2	FIP	TTCGCCCCCAGTTCAGAGTGAGTCAGGCACTGTCTGAAACT	41	[25]
	BIP	TGCTTCCGGAGTATCGGGGAGCAGTCCCCAGTATCGCTGA	40	
	F3	CGCTCTCAGGCAGATACAGAG	21	
	B3	CCCCCTGATGATGGCAATT	19	
	FL	GCGTCATCGTATACACAGGAGC	22	
	BL	GATGGTGTCAGAGTGGGGAGAA	22	

### Optimization of LAMP assay

EPEC isolate was initially re-confirmed by PCR. Subsequently, the LAMP assay based on the initial reaction condition was performed to verify the functionality of the designed lamp primers. The amplification result was evaluated by using: Turbidity assessment, agarose gel electrophoresis (and SYBR Green I dye detection methods (Supplementary Fig. 1). The stepwise optimization process was performed for the identification of the most effective combination of parameters to enhance reaction efficiency and specificity of the assay. The detailed optimization process and corresponding results for each factor are presented below, providing clear evidence and justification for the final selected reaction conditions. Once the optimal reaction condition of the test factor was determined, it was used in the subsequent experiments. Strict contamination control procedures were used in the present study to reduce the possibility of aerosol contamination and carryover throughout the LAMP workflow. These precautions included using aerosol-resistant pipette tips, keeping pre- and post-amplification areas physically apart, and workflow procedures were strictly followed.

#### Optimization of MgSO4 concentration

The range of MgSO4 concentration from 2.0 to 18 mm were evaluated and LAMP products showed green color in SYBR Green I staining at the concentration range from 4.0 to 12.0 mM indicating the presences of amplification and the appearance of orange color at the concentration of 2.0 mM of Mgso_4_ indicates the absence of amplification (Supplementary Fig. 2a). In agarose gel electrophoresis, the LAMP products displayed as a ladder-like bands from the reaction with the Mgso_4_ concentrations ranging from 4.0 to 18.0 mM. The brightest band was appeared at 10.0 mM, indicating it is the optimal MgSO₄ concentration for the reaction (Supplementary Fig. 2b).

#### Optimization of temperature

During the evaluation of LAMP assay under different temperature ranging from 57 to 65 °C, a fluorescent green color was displayed at all temperature ranges after SYBR Green I staining (Supplementary Fig. 3a) and the typical ladder-like bands were observed in all nine different reaction temperatures in agarose gel. The robust band was seen at 62 °C (Supplementary Fig. 3b). Therefore, 62 °C regarded as an optimal temperature for the LAMP reaction.

#### Optimization of incubation time

Different time interval ranging from 10 up to 70 min was evaluated to determine optimal time for LAMP reaction. Until 35 min, no color change was displayed by SYBR green I staining, it remained orange, starting from 40 up to 65 min a fluorescent green color was displayed after SYBR Green I staining indicates the positive result and at 70 min no color change was observed (Supplementary Fig. 4a). In agarose gel electrophoresis, the LAMP products displayed ladder-like bands starting from 40 up to 65 min. No ladder-like bands were seen between 10 min up to 35 min and 70 min. The brightest band was produced at 60 min, indicating the optimal time for LAMP reaction was 60 min (Supplementary Fig. 4b).

#### Optimization of Bst polymerase concentration

The concentrations of bst polymerase ranging from 2-16U were evaluated. A fluorescent green color was displayed after SYBR Green I staining in all evaluated concentration of the bst polymerase, indicating the presence of amplification (Supplementary Fig. 5a). The robust band was displayed at 8.0 U of bst polymerase. Therefore, 8U of bst polymerase was considered as an optimal concentration for the reaction (Supplementary Fig. 5b).

#### Optimization of DNTPs concentration

All LAMP products from nine different concentration of dNTPs ranging from 0.4 to 2 mM showed a fluorescent green color in SYBR Green I staining (Supplementary Fig. 6a). Agarose gel electrophoresis result shows typical ladder-like bands in all evaluated dNTPs concentration, LAMP products showed strong bands in reactions with 1.4 mM dNTPs, indicating the optimal concentration of dNTPs for the LAMP reaction was 1.4 (Supplementary Fig. 6b).

#### Application of optimized LAMP protocol for EPEC detection

**Fig. 1:**
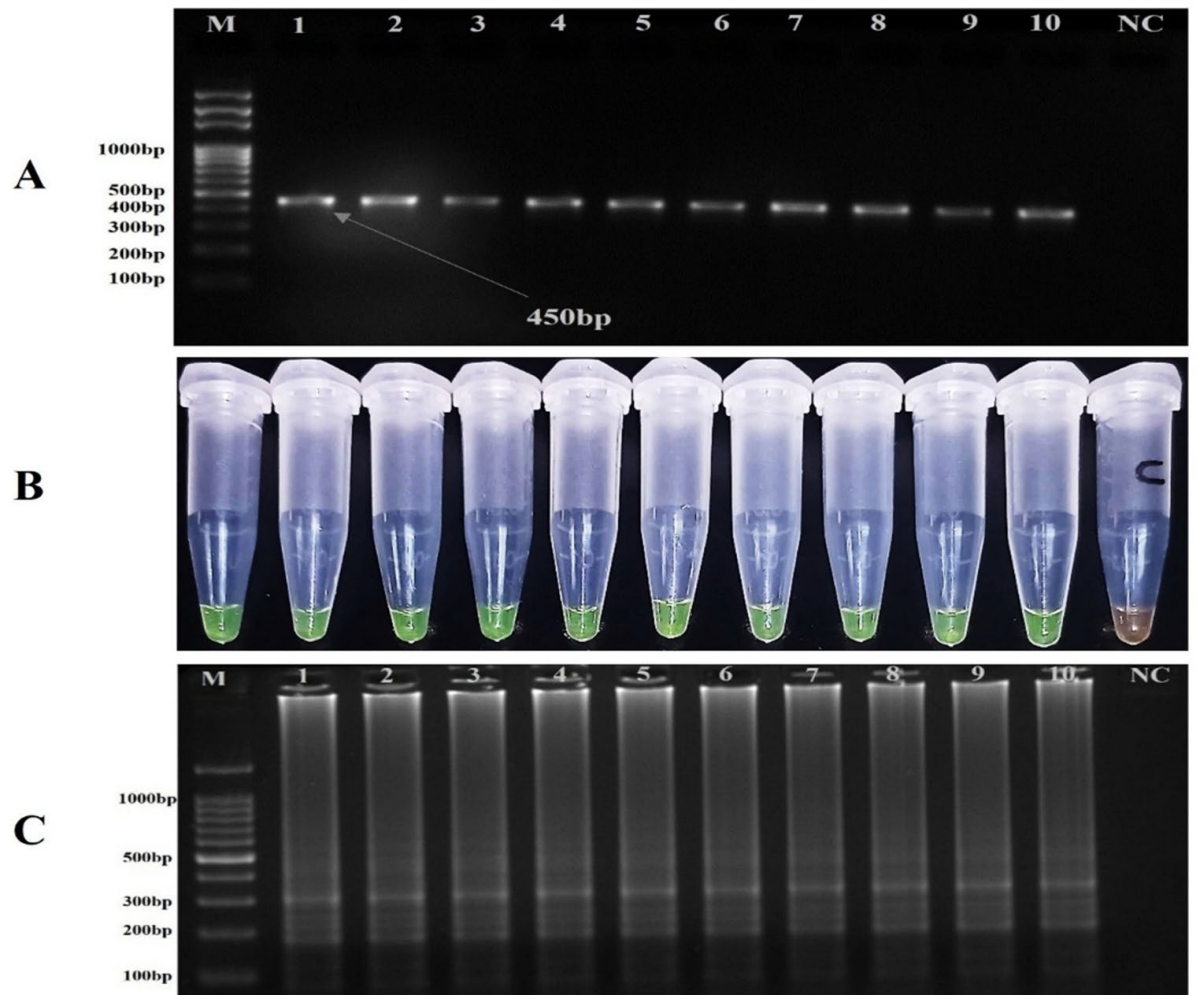
PCR and LAMP amplification of 10 EPEC strain: **(A)** PCR amplification product of 10 EPEC isolates product size was 450 bp; lane M: DNA ladder (100plus), lane 1: 46 A, lane2:C89, lane3: C75, lane 4:C71, lane 5:CF 1, lane 6:CF31, Lane 7: EV96, lane8: IH 31, lane 9: CF 28, lane 10: IH10, Tube 11: negative control (ddwater). **(B) **SYBR green I detection of Lamp product of EPEC amplification; Tube 1–10 shows a positive result: Tube 1: 46 A, Tube 2:C89, Tube 3: C75, Tube 4:C71, Tube 5:CF 1, Tube 6:CF31, Tube 7: EV96, Tube 8: IH 31, Tube 10: CF 28, Tube11: IH10, lane 12: negative control (ddwater). **C)** Gel electrophoresis detection of LAMP product of EPEC isolates shows ladder like appearance; lane1: DNA ladder (100plus), lane 2: 46 A, lane3:C89, lane4: C75, lane 5:C71, lane6:CF 1, lane 7:CF31, Lane 8: EV96, lane9: IH 31, lane 10: CF 28, lane 11: IH10, lane 12: negative control (ddwater)

After comprehensive evaluation of five critical factors, the optimal LAMP reaction conditions were established as 2.5 µL of isothermal amplification buffer, 10 mM MgSO4 solution, 1.4 mM dNTPs, 1.8 mM FIP and BIP, 0.4 mM F3 and B3, 0.2 mM LB, 8.0 U Bst polymerase, and 2.0 µL of the target DNA template are all included in the final LAMP reaction. For 60 min, the reaction mixture was incubated at 62 C^0^. Ten EPEC strains were subjected to a subsequent LAMP reaction under this optimized condition, which was verified by PCR (Fig. 1a).Successful amplification of LAMP assay was verified by SYBR Green I staining, with positive reactions producing a visible green color (Fig. 1b) and further confirmed by agarose gel electrophoresis, which revealed the characteristic of ladder-like bands of LAMP products (Fig. 1c).

Figure 1: PCR and LAMP amplification of 10 EPEC strain: **(A)** PCR amplification of 10 EPEC isolates eae gene product size was 450 bp; lane M: DNA ladder (100plus), lane 1: 46 A, lane2:C89, lane3: C75, lane 4:C71, lane 5:CF 1, lane 6:CF31, Lane 7: EV96, lane8: IH 31, lane 9: CF 28, lane 10: IH10, Tube 11: negative control (ddwater). **(B)**SYBR green I detection of Lamp product of EPEC amplification; Tube 1–10 shows a positive result: Tube 1: 46 A, Tube 2:C89, Tube 3: C75, Tube 4:C71, Tube 5:CF 1, Tube 6:CF31, Tube 7: EV96, Tube 8: IH 31, Tube 10: CF 28, Tube11: IH10, lane 12: negative control (ddwater). **C)** Gel electrophoresis detection of LAMP product of EPEC isolates shows ladder like appearance; lane M: DNA ladder (100plus), lane 1: 46 A, lane 2:C89, lane 3: C75, lane 4:C71, lane 5:CF 1, lane 6:CF31, Lane 7: EV96, lane 8: IH 31, lane 9: CF 28, lane 10: IH10, lane 11: negative control (NC).

### Validation of LAMP assay

PCR was used in parallel with LAMP to validate the LAMP assay by using 60 bacterial isolates (Table 3). The isolates were considered as true negative when they were not EPEC, and true positive when they were EPEC, based on the results of the PCR. Test negative indicates that the isolate was negative in the LAMP test, whereas test positive indicates that the isolate was positive by the LAMP assay. A 2 × 2 contingency matrix was utilized to assess the LAMP assay’s sensitivity, specificity, and overall performance (Supplementary Table 1).

**Table 3:** Comparative performance of LAMP and PCR assays based on parallel testing of isolates

N*o*	Isolate code	Bacterial strain	PCR eae	PCR stx1	PCR stx2	LAMP eae	LAMP stx1	LAMP stx2
1	46 A	EPEC	**+**	**-**	**-**	**+**	**-**	**-**
2	C89	EPEC	**+**	**-**	**-**	**+**	**-**	**-**
3	C75	EPEC	**+**	**-**	**-**	**+**	**-**	**-**
4	C71	EPEC	**+**	**-**	**-**	**+**	**-**	**-**
5	CF10	EPEC	**+**	**-**	**-**	**+**	**-**	**-**
6	CF31	EPEC	**+**	**-**	**-**	**+**	**-**	**-**
7	EV96	EPEC	**+**	**-**	**-**	**+**	**-**	**-**
8	IH 31	EPEC	**+**	**-**	**-**	**+**	**-**	**-**
9	CF28	EPEC	**+**	**-**	**-**	**+**	**-**	**-**
10	IH 10	EPEC	**+**	**-**	**-**	**+**	**-**	**-**
11	024	EAEC	-	**-**	**-**	-	**-**	**-**
12	CF71	EAEC	-	-	-	-	-	-
13	CB 82	EAEC	-	-	-	-	-	-
14	IH 64	EAEC	-	-	-	-	-	-
15	EV71	EAEC	-	-	-	-	-	-
16	IH80	EAEC	-	-	-	-	-	-
17	61 C	EAEC	-	**-**	**-**	-	**-**	**-**
18	72I	EAEC	-	**-**	**-**	-	**-**	**-**
19	7I	EAEC	-	**-**	**-**	-	**-**	**-**
20	73I	EAEC	-	**-**	**-**	-	**-**	**-**
21	CF10	ETEC	-	**-**	**-**	-	**-**	**-**
22	73 A	ETEC	-	**-**	**-**	-	**-**	**-**
23	C105	ETEC	-	**-**	**-**	-	**-**	**-**
24	C106	ETEC	-	**-**	**-**	-	**-**	**-**
25	C114	ETEC	-	**-**	**-**	-	**-**	**-**
26	C104	ETEC	-	-	-	-	-	-
27	C99	ETEC	-	-	-	-	-	-
28	C102	ETEC	-	-	-	-	-	-
29	C95	ETEC	-	-	-	-	-	-
30	C93	ETEC	-	-	-	-	-	-
31	C19	EHEC	**+**	**+**	**-**	**+**	**+**	**-**
32	C39	EHEC	**+**	**-**	**+**	**+**	**-**	**+**
33	C46	EHEC	**+**	**-**	**+**	**+**	**-**	**+**
34	C141	EHEC	**+**	**-**	**+**	**+**	**-**	**+**
35	EV95	STEC	**-**	**+**	**-**	**-**	**+**	**-**
36	IH 85	STEC	**-**	**+**	**+**	**-**	**+**	**+**
37	IH 86	STEC	**-**	**+**	**+**	**-**	**+**	**+**
38	S04	STEC	**-**	**+**	**+**	**-**	**+**	**+**
39	C18	STEC	**-**	**-**	**+**	**-**	**-**	**+**
40	C12	STEC	**-**	**+**	-	-	**+**	**-**
41	IH 12	STEC	-	**+**	**+**	-	**+**	**+**
42	C35	STEC	-	-	**+**	-	-	**+**
43	CF 49	STEC	-	**+**	**+**	-	**+**	**+**
44	S04	STEC	-	**+**	**+**	-	**+**	**+**
45	C57	EIEC	-	**-**	**-**	-	**-**	**-**
46	C30	EIEC	-	**-**	**-**	-	**-**	**-**
47	C63	Nonpathogenic *E. coli*	-	**-**	**-**	-	**-**	**-**
48	C68	Nonpathogenic *E. coli*	-	**-**	**-**	-	**-**	**-**
49	C38	Nonpathogenic *E. coli*	-	**-**	**-**	-	**-**	**-**
50	C40	Nonpathogenic. *coli*	-	**-**	**-**	-	**-**	**-**
51	C126	Nonpathogenic *E. coli*	-	**-**	**-**	-	**-**	**-**
52	C82	Nonpathogenic *E. coli*	-	-	-	-	-	-
53	C 137	Nonpathogenic *E. coli*	-	-	-	-	-	-
54	C142	Nonpathogenic. *coli*	-	-	-	-	-	-
55	C143	Nonpathogenic. *coli*	-	-	-	-	-	-
56	CF 45	Nonpathogenic *E. coli*	-	-	-	-	-	-
57	P 1	*Pseudomonas*	-	-	-	-	-	-
58	K28	*Klebsiella pneumoniae*	-	-	-	-	-	-
59	SA2	*Staphylococcus aureus*	-	-	-	-	-	-
60	S23	*Salmonella typhi*	-	-	-	-	-	-

#### Specificity of the LAMP assay

60 bacterial isolates comprising 10 EPEC, 46 non-EPEC *E. coli* strains, and 4 non-E. coli bacterial species were used to assess the specificity of the LAMP assay. The LAMP test revealed that none of the 50 non-EPEC bacterial isolates had false-positive results. No amplification was observed in the non-*E. coli* species, confirming the assay’s genus-level specificity and absence of cross-reactivity (Supplementary Fig. 7). Although the amplification of the eae gene was detected in several EHEC isolates, only those lacking the *stx1* and *stx2* genes were classified as EPEC. This genotypic distinction allows a clear differentiation of EPEC and EHEC strains, ensuring accurate identification of EPEC isolates. EHEC isolate that contains eae gene amplification result shown in (Supplementary Fig. 8). The absence of false positives among the 50 non-EPEC isolates confirms the assay’s 100% analytical specificity (Table 4).

#### Sensitivity of the LAMP assay

The developed LAMP assay exhibits great specificity on a limited strain panel and has a software-calculated sensitivity of 100% by detecting all 10 EPEC strains. Additionally, the study revealed a positive predictive value of 100%, indicating that every sample testing positive by the LAMP assay was conclusively identified as an EPEC isolate. Similarly, a negative predictive value of 100% was observed, confirming that all negative results demonstrated accurately the absence of EPEC. These findings reflect the perfect agreement between the LAMP assay results and the true status of all tested samples (Table 4).

**Table 4:** Statistical analysis of the diagnostic performance of the LAMP assay using MedCalc version 23.2.1 software

Statistic	Formula	Value	95%CI
Sensitivity	(TP/(TP + FN))100%	100.000%	69.150% to 100.000%
Specificity	(TN/(TN + FP))100%	100.000%	92.888% to 100.000%
Positive predictive value	(TP/(TP + FP))100%	100.000%	69.150% to 100.000%
Negative predictive value	(TN/(TN + FN))100%	100.000%	92.888% to 100.000%
Accuracy	(TP + TN)/(TP + TN +FP + FN) 100%	100.000%	94.037% to 100.000%

#### Cohen’s kappa test statistics

The Cohen’s Kappa test statistic(K) was used to evaluate the agreement between the LAMP and PCR assay. The result showed perfect agreement with a Kappa value of 1.00, which indicates, the LAMP and PCR tests showed a perfect agreement on tested isolates (Table 5).

K = 1 Shows a perfect agreement between PCR and LAMP assay.

**Table 5:** Agreement analysis using Cohen’s kappa statistic (κ)

LAMP	PCR				Weighted Kappa	95%CI
	Test	Positive	Negative	Total		
	Positive	10	0	10	1.0	1.00 to 1.00
	Negative	0	50	50		
	Total	10	50	60		

### Lowest detection limit of LAMP assay (LOD)

#### Lowest detection limit of LAMP assay DNA concentration/reaction

**Fig. 2:**
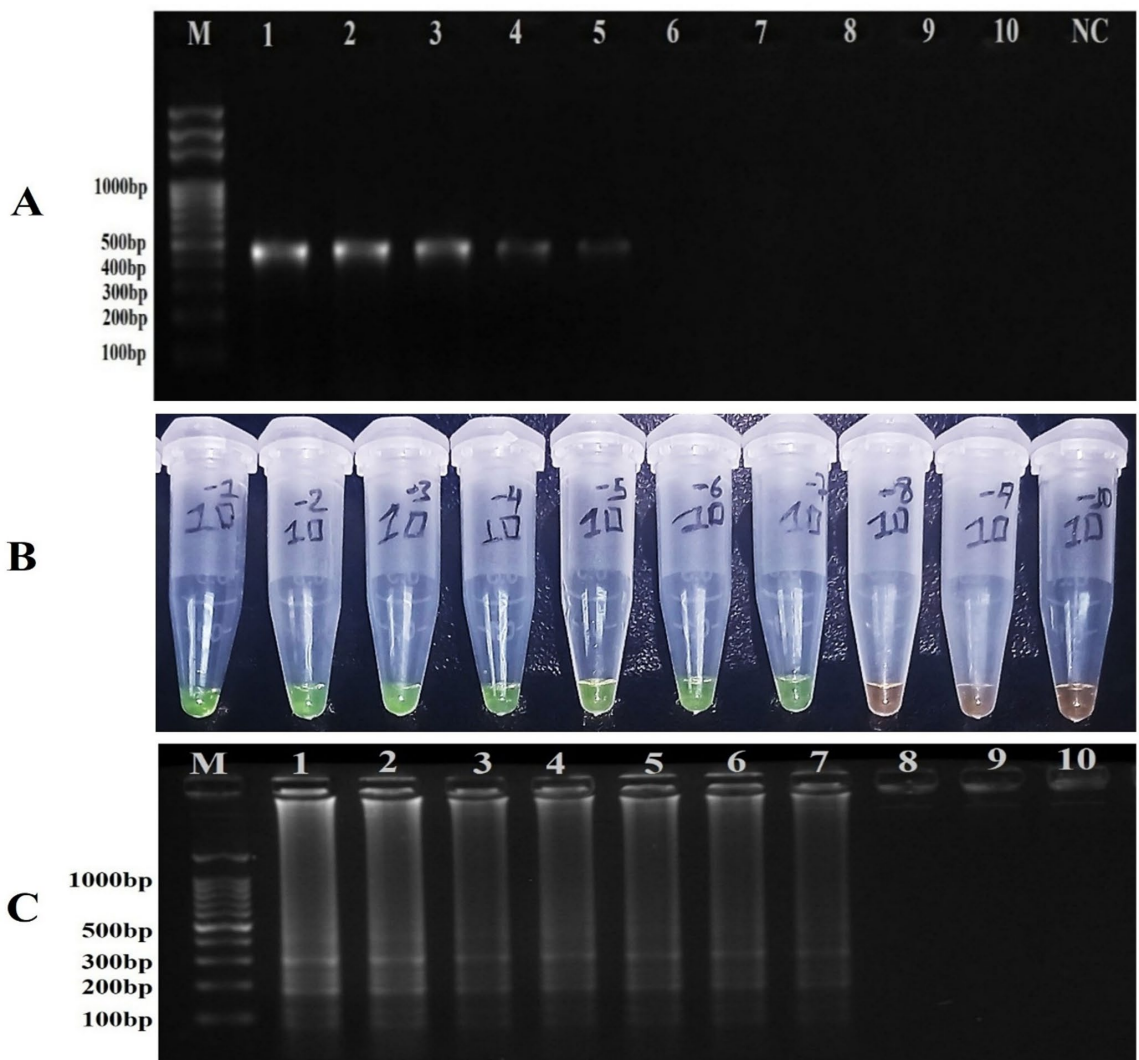
Lower detection limit comparison of LAMP and PCR assays using 10-fold serial dilutions of EPEC DNA: **(A)** Gel electrophoresis visualization of PCR’s lower detection limit. Lane M: DNA ladder, lane 1: 10^− 1^, lane 2: 10^− 2^, lane 3: 10^− 3^, lane 4: 10^− 4^, lane 5: 10^− 5^, lane 6:10^− 6^, lane 7: 10^− 7^, lane 8: 10^− 8^, lane 9: 10 − ^9^ lane 10: 10^–10,^ Lane NC: negative control. **(B)** LAMP assay detection limit result comparison by SYBR green I detection: Tube 1: 10^− 1^, tube 2: 10^− 2^, tube 3: 10^− 3^, tube 4: 10^− 4^, tube 5: 10^− 5^, tube 6:10^− 6^, tube 7: 10^− 7^, tube 8: 10^− 8^, tube 9: 10 − ^9^, tube10: 10^–10^. **(C)** Lower detection limit of LAMP assay analyzed by gel electrophoresis. Lane M: DNA ladder, lane 1: 10^− 1^, lane 2: 10^− 2^, lane 3: 10^− 3^, lane 4: 10^− 4^, lane 5: 10^− 5^, lane 6:10^− 6^, lane 7: 10^− 7^, lane 8: 10^− 8^, lane 9: 10 − 9, lane 10: 10^–10^, Lane NC: negative control

Detection limit of the developed LAMP assay was determined by performing a series of 10-fold serial dilutions of EPEC template DNA under optimal reaction conditions. No positive reaction was observed using less than 0.05pg of DNA per reaction, indicating the lowest detection limit of the LAMP assay was 0.05pg per reaction. In contrast the conventional PCR showed a lowest detection limit of 5pg (Fig. 2). The detection outcomes for both LAMP and PCR across the dilution series are summarized in (Supplementary Table 2).

#### Lowest detection limit of LAMP assay in terms of CFU/ml and CFU /g of stool

The number of bacterial cells detected by the developed LAMP test was calculated using the standard plate counting method. Using the plate-counting approach, the undiluted culture’s total viable count was 7 × 109CFU/mL. There is a noticeable difference in the detection limits between the LAMP and PCR assay results. Particularly, compared to 7 × 10³ CFU/mL for conventional PCR, the LAMP assay showed noticeably greater sensitivity, with a detection limit as low as 70 CFU/mL. This indicates that detecting the target EPEC in pure culture, the developed LAMP assay is around 100 times more sensitive than PCR. Additionally, the lowest LAMP detection limit in a spiked stool sample was 7 × 10^2^ cfu/g stool. On the other hand, 7 × 10^4^ cfu/g stool for PCR (Table 6).

**Table 6:** Comparative detection limits of LAMP and PCR in terms of CFU/ml and CFU/g

Dilution Factor	EPEC dilution CFU/ml	LAMP	PCR	Spiked Stool samples CFU /g	LAMP	PCR
10^− 0^	7 × 10^9^ CFU/mL	**+**	**+**	7 × 10^9^ CFU/g	**+**	**+**
10^− 1^	7 × 10^8^ CFU/mL	**+**	**+**	7 × 10^8^ CFU/g	**+**	**+**
10^− 2^	7 × 10^7^ CFU/Ml	**+**	**+**	7 × 10^7^ CFU/g	**+**	**+**
10^− 3^	7 × 10^6^ CFU/mL	**+**	**+**	7 × 10^6^ CFU/g	**+**	**+**
10^− 4^	7 × 10^5^ CFU/mL	**+**	**+**	7 × 10^5^ CFU/g	**+**	**+**
10^− 5^	7 × 10^4^ CFU/mL	**+**	**+**	7 × 10^4^ CFU/g	**+**	**+**
10^− 6^	7 × 10^3^ CFU/mL	**+**	**+**	7 × 10^3^ CFU/g	**+**	**-**
10^− 7^	7 × 10^2^ CFU/mL	**+**	-	7 × 10^2^ CFU/g	**+**	-
10^− 8^	7 × 10^1^ CFU/mL	**+**	-	7 × 10^1^ CFU/g	-	-
10^− 9^	7 × 10^0^ CFU/mL	-	-	7 × 10^0^ CFU/g	-	-
10^− 10^	7 × 10^− 1^ CFU/mL	-	-	7 × 10^− 1^ CFU/g	-	-

## Discussion

The global health system would greatly benefit from an accurate, rapid and easy methods to diagnose pathogenic microorganisms. In particular diarrheal diseases remain a significant challenge globally. The burden of this infection indicates the necessity of efficient diagnostic tools. LAMP is a cutting-edge molecular test that meets the demand for quick, precise, and reasonably priced diagnosis. Numerous studies have effectively demonstrated LAMP’s remarkable contribution to filling the gap in molecular diagnostics. It has been successful in identifying bacterial, viral, and parasite diseases [26–29]. Recently, LAMP assays have been developed for various diarrheal pathogens including pathogenic *Escherichia coli* strains such as enterohemorrhagic *Escherichia coli*, enterotoxigenic *Escherichia coli*, Enteroaggregative *Escherichia coli* and Shiga toxin producing *Escherichia coli* [30–33]. In addition, LAMP has been effectively used for the detection of other diarrheal pathogens including *Shigella flexneri*, *clostridium difficile*, *Vibrio cholerae*,* Giardia lamblia*, *Entamoeba histolytica* and *rotavirus* [34–39].

In this study we developed loop mediated isothermal amplification assay for a rapid, sensitive and resource friendly detection of Enteropathogenic *Escherichia coli* (EPEC*)*. The eae gene was used as a target gene for LAMP primer designing. Despite being a crucial marker for EPEC identification, the eae gene is not exclusive to EPEC; it may also be found in EHEC/STEC and other attaching-and-effacing *E. coli* pathotypes. To enhance the specificity and accuracy of the developed assay, stx1 *and* stx2 lamp primers are used along with eae primer, this multiplex approach enabled precise detection of EPEC from other closely related pathotypes, thereby enhancing the reliability of the detection system. The specificity of the developed LAMP assay was evaluated to confirm the assay’s ability for distinguishing EPEC from other closely related organisms. In comparison to previous study where the LAMP assay shows 100% sensitivity and 97.05% specificity for detecting enterohemorrhagic *Escherichia coli* (EHEC) [40], the present study showed both sensitivity and specificity of 100% for EPEC detection, reflecting perfect accuracy in identifying true positive and true negative samples. The developed LAMP assay detected all 10 tested EPEC-positive samples, a software-calculated sensitivity was 100%. While these results indicate promising performance under the tested conditions, the small sample size means that further studies with a larger number of samples are needed to fully assess the assay’s reproducibility and sensitivity.

The agreement between the developed LAMP assay and PCR was measured by Cohen’s kappa statistic (k = 1) and the result indicates perfect agreement between PCR and LAMP. This level of agreement is higher than previously reported LAMP assays for the detection of EHEC [40]. Three repeat measures of reproducibility testing on a subset of ten samples revealed perfect concordance (kappa = 1.0), which supports the consistency of the assay under repeated testing. However, this reproducibility analysis was limited to a small subset of samples. Therefore, large scale reproducibility and blinded validation studies are recommended to further validation of the developed LAMP Assay. The present study demonstrates the superior sensitivity of the LAMP assay in comparison to conventional PCR for the detection of EPEC. Conventional PCR demonstrated a lower detection limit of 5 pg per reaction, whereas the new LAMP test could detect DNA at a concentration as low as 0.05 pg per reaction. This suggests that the new LAMP test has a sensitivity of about 100 times that of PCR. The outcomes are in line with earlier LAMP assays that have a 50 fg/reaction detection limit and a 5 pg/reaction PCR detection limit for the identification of *Mycobacterium bovis* target DNA [41].

The developed LAMP technique demonstrated a 100-fold higher sensitivity than traditional PCR in detecting viable bacterial cells, detecting as low as 70 CFU/mL of EPEC in pure culture as opposed to 7 × 10³ CFU/mL by PCR. Similarly, the LAMP found 7 × 10² CFU/g in spiked stool samples, but PCR needed 7 × 10² CFU/g. The presence of inhibitors, such as bile salts and plant-based polysaccharides frequently present in fecal samples, may be the cause of the sensitivity differential between the fecal sample and pure culture [42]. A study on diarrheal illnesses found that 88 (66%) of 134 children had bacterial, viral, or parasitic pathogens as their etiologic agent. Diarrheagenic E. coli was found in 42 (31%) of these children, with enteric adenovirus (10%), Salmonella (10%), Campylobacter (9%), Giardia (6%), rotavirus (4%), and Cryptosporidium (2%) following in order. 30 (71%) of the 42 *E. coli* isolates were EPEC;6 (14%) were STEC, 3 were EHEC; 4 (10%) were EAEC; 1 (2%) was (ETEC); and 1 (2%) was (EIEC) [43].

Among diarrheal cases where EPEC was the pathogen detected, the bacterial load was around 2.9 × 10⁶ CFU/g [44]. Therefore, the developed LAMP assay is valuable for direct patient diagnosis, as it enables a sensitive detection of EPEC from stool sample, with a lower detection limit of 7 × 10² CFU/g. In order to assess the analytical performance of the LAMP assay, all experiments were performed using spiked stool samples due to limited availability of clinical specimens. The assay demonstrated high analytical sensitivity and specificity, and we anticipate that implementing an efficient stool DNA extraction method to remove inhibitory substances to further enhance the assay’s sensitivity, pending further clinical validation. EPEC may be present in stool due to asymptomatic carriage, past infection or co-infection with other diarrheal pathogens. Therefore, LAMP results should be interpreted alongside clinical correlation. Furthermore, the assay may have potential for epidemiological studies and outbreak detection from stool samples with pending further validation in field and clinical settings. Compared to conventional PCR, LAMP generate the results in 60 min, compared to the 1.5 to 3 h needed for PCR experiments, this is significantly faster. Additionally, LAMP is resource friendly, it requires less expensive equipment and simpler procedures. Therefore, it can be used in low-resource environments and for point-of-care testing [45]. Despite LAMP’s speed and sensitivity, the small-sized and complex amplification products it produces may restrict its specificity and raise the possibility of interpretation errors when depending on endpoint detection techniques. Thus, to guarantee accurate result interpretation, thorough assay optimization, suitable controls, and adequate product detection method designing are necessary [46–49].

## Conclusion

LAMP is a rapid, simple, sensitive, and specific molecular diagnostic method. In this study, we developed and optimized the LAMP assay for the detection of EPEC. The assay was evaluated using a limited panel of bacterial strains and showed high analytical sensitivity and specificity under the tested conditions. With further validation using a larger and more diverse set of clinical samples, the developed assay may contribute for rapid and accessible alternative diagnostics method, particularly in resource-limited settings where standard molecular diagnostics are less readily available.

## Supplementary Information

Below is the link to the electronic supplementary material.

Supplementary Material 1

## Data Availability

Data Availableupon request.

## Abbreviations

EPEC: Enteropathogenic Escherichia coli
TP: True positive
TN: True negative
NPV: Negative predictive value
PPV: positive predictive value
eae: E. coli attaching and effacing gene
Stx: Shiga toxin encoding gene
Bfp: bundle forming protein
A/E: attaching and effacing
LF: Loop Forward Primer
LB: Loop Backward Primer
FIP: Forward inner Primer
BIP: Backward Forward Primer
F3: Forward outer Primer
B3: Backward Outer Primer

## Declarations

### Tesfaye Sisay tessema

Conceptualization, Supervision, Validation, Writing and Editing.
