# Supplementary material for: Development loop-mediated isothermal amplification assay for detection of enteropathogenic Escherichia coli (EPEC)
Source: Mol Biol Rep. 2026 Jun 26;53(1):1010. doi: 10.1007/s11033-026-12206-x (PMC13309408; doi:10.1007/s11033-026-12206-x)
Supplement: Supplementary file 1 — Supplementary Material 1 [file 11033_2026_12206_MOESM1_ESM.docx]

**Supplementary file:**

Development Loop-Mediated Isothermal Amplification assay for detection of Enteropathogenic *Escherichia coli* (EPEC)

Alazar Amare Amdiyee ^1,2^, Tesfaye Sisay Tessema^1*^

1. Addis Ababa University, biotechnology research center, Addis Ababa, Ethiopia.

## Dilla University, College of Medicine and Health Science, Department of Medical Laboratory Sciences, Dilla, Ethiopia.

^*^Corresponding author; Alazar Amare Amidyee; email address: amarealazar633@gmail.com


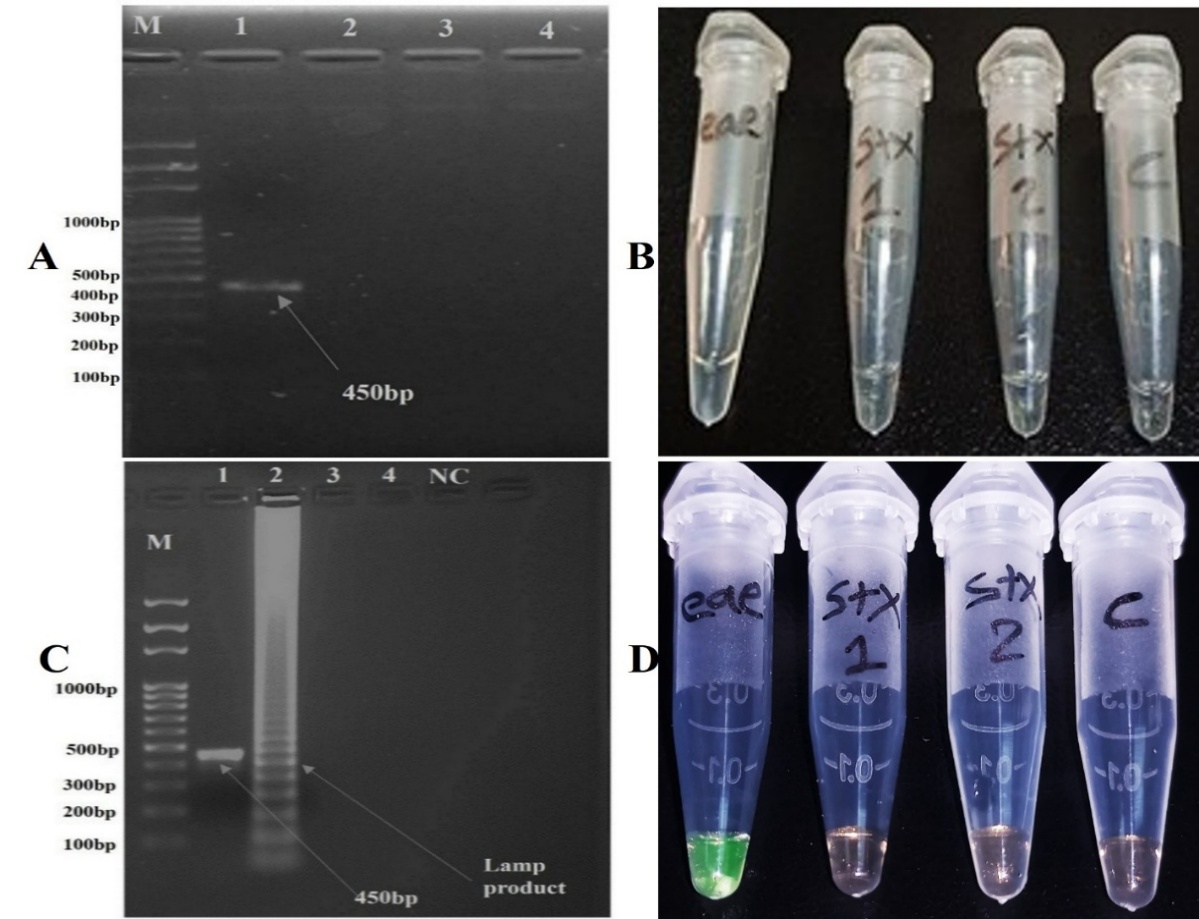


Supplementary Figure 1: LAMP and PCR based Detection of EPEC **A**) Shows agarose gel electrophoresis PCR product: Lane M=100 bp marker, lane 1= PCR product of eae gene, Lane2: Stx1, Lane 3: stx2, lane 4: Negative Control(NC); **B**) Visual turbidity assessment of LAMP Product; **C**) Agarose gel electrophoresis based detection of LAMP product: Lane M=100 bp marker, lane 1= PCR product of EPEC, Lane 2= eae LAMP product, lane 3: stx1 -, lane 4: stx2**-; D**) SYBR Green I based detection of LAMP product.


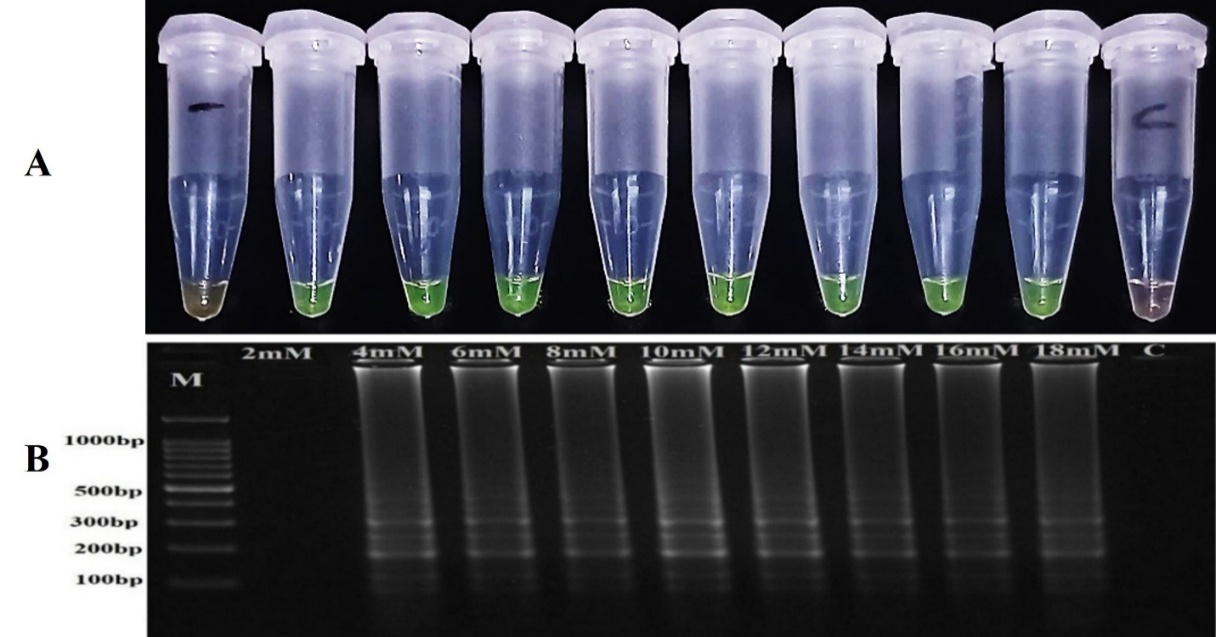


Supplementary Figure 2: LAMP reactions with different concentrations ranging from 2.0 to 18 mm of MgSO4 solution **(A).** Assessments of LAMP products based on SYBR Green I visualization **(B).** Agarose gel electrophoresis result.


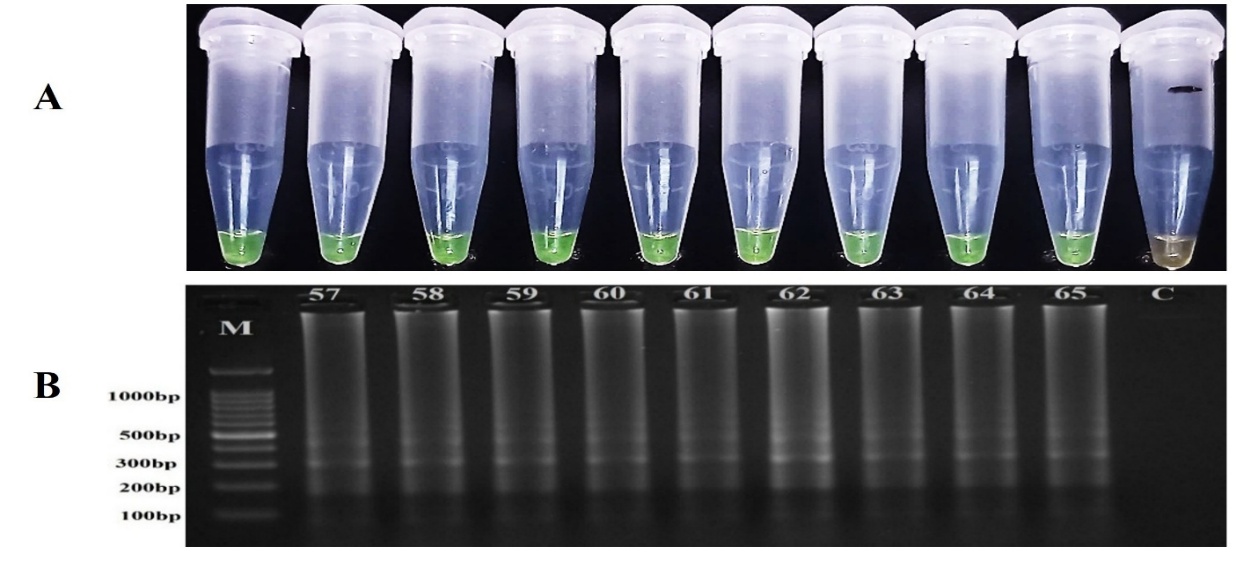


Supplementary Figure 3: Results of the LAMP reactions at different a temperature of 57,58,59,60,61,62,63,64,65. A) SYBR green I detection. **B)** Agarose gel electrophoresis results.


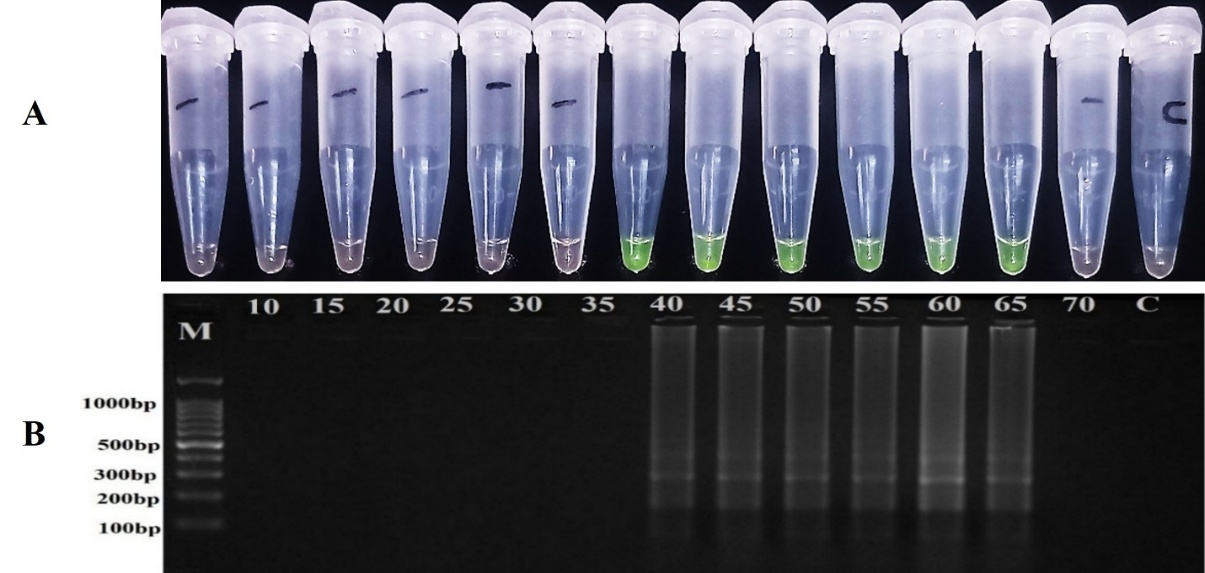


Supplementary Figure 4: LAMP amplification with different reaction times10, 15, 20, 25, 30, 35,40,45,50, 55,60,65, and 70 min. A) SYBR Green I visualization. **B)** Agarose gel electrophoresis result.


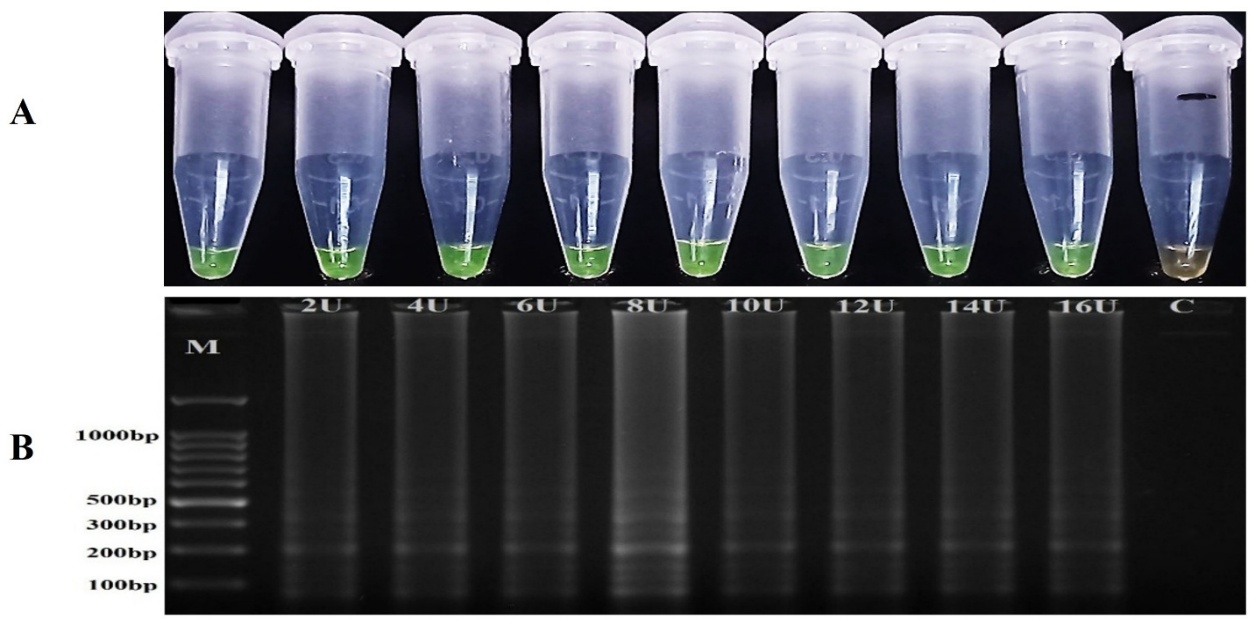


Supplementary Figure 5: Result of LAMP reactions with different Bst polymerase concentrations 2.0, 4.0, 6.0, 8.0, 10.0, 12.0, 14.0 ,16.0U, respectively. **A)** SYBR Green I visualization. **(B)** Agarose gel electrophoresis result.


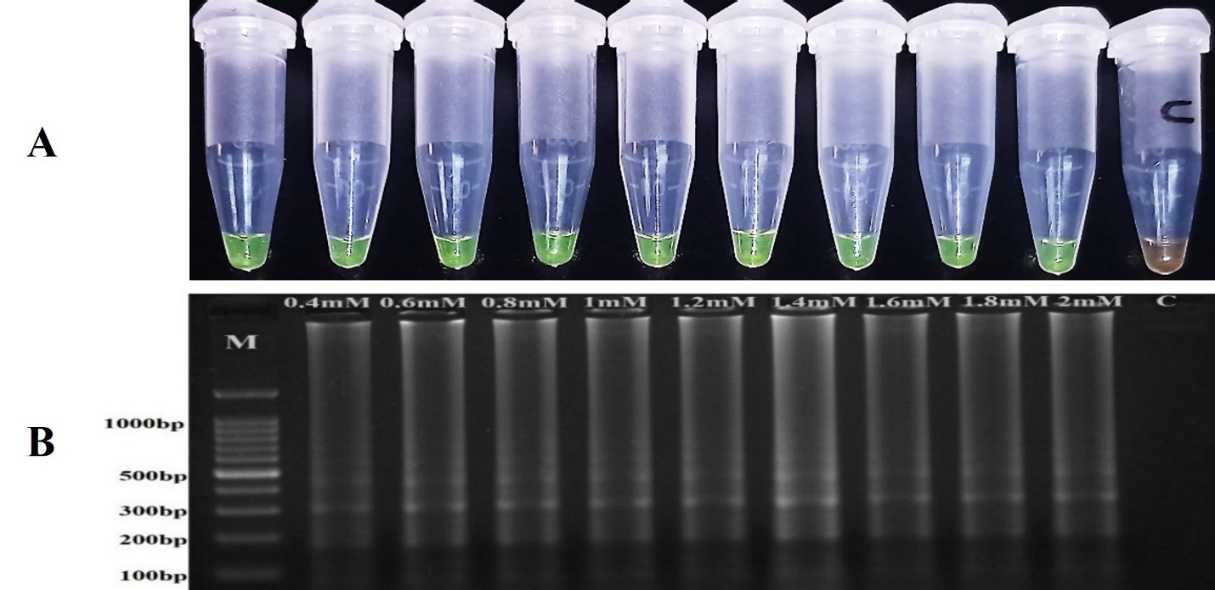


Supplementary Figure 6: Result of LAMP reactions with different dNTPS concentrations ranging from 0.4, 0.6, 0.8, 1.0, 1.2, 1.4 ,1.6,1.8,2 respectively**. A)** Shows SYBR Green I visualization. **B)** Shows agarose gel electrophoresis result.


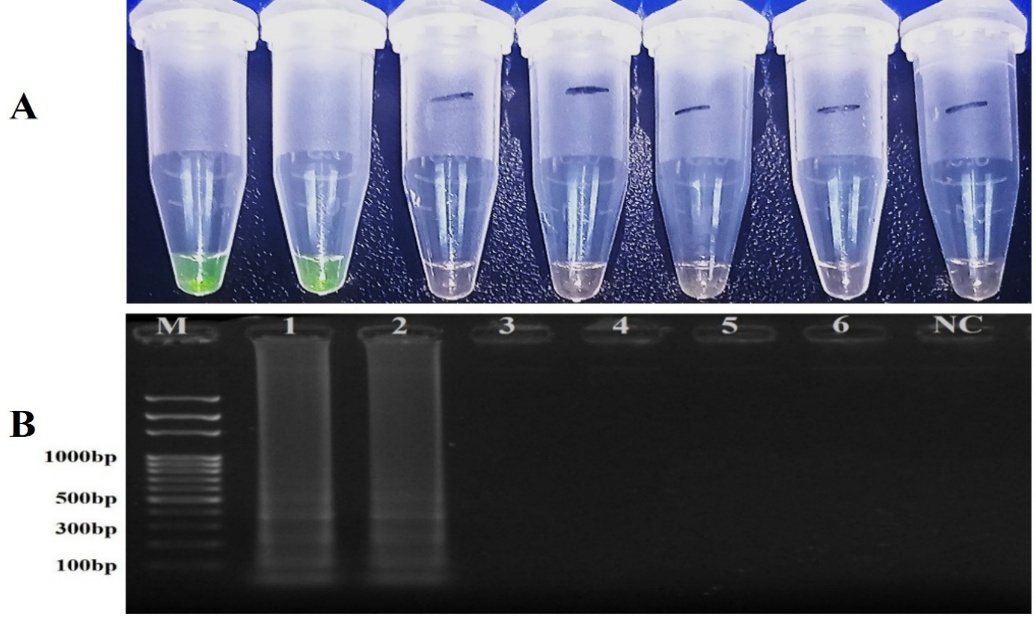


Supplementary Figure 7: Specificity evaluation of the method on non-E. coli bacteria: **(A)** SYBR green I detection; Tube 1: 46A(EPEC), Tube 2:C89(EPEC), Tube 3: klebsiella pneumonia, Tube 4: pseudomonas, Tube 5: Staphylococcus aureus, Tube 6: Salmonella, Tube 7: negative control. **B)** Gel electrophoresis result; laneM: DNA ladder (100plus), lane 1: 46A, lane2:C89, lane3: klebsiella pneumonia, lane 4: pseudomonas: lane5: Staphylococcus aureus, lane 6: salmonella, Lane 7: negative control (ddwater).


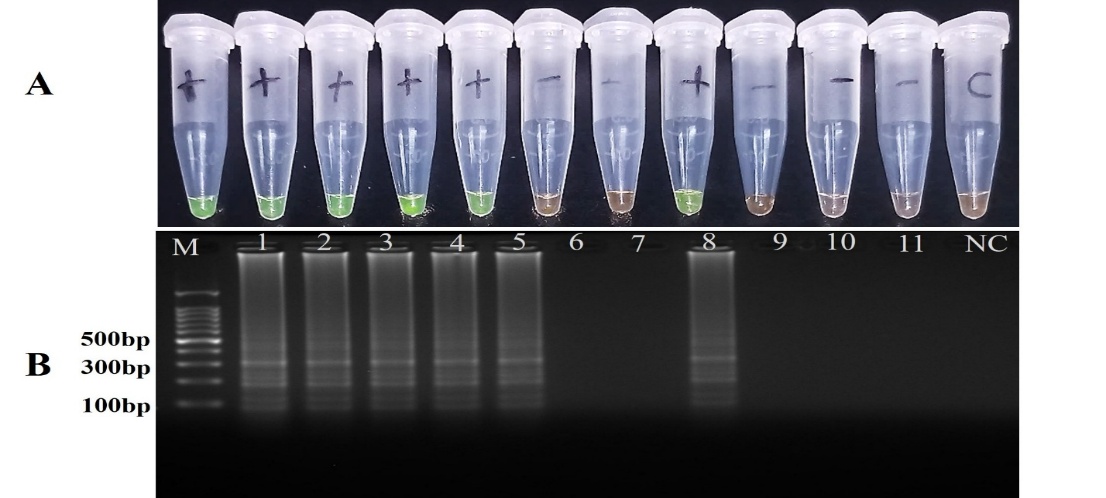


Supplementary Figure 8: Evaluation of the LAMP assay’s specificity on non-EPEC E. coli: **(A)** SYBR green I detection; Tube 1: 46A (EPEC), Tube 2:C89, Tube 3: C75, Tube 4: C71, Tube 5: CF10, Tube 6: 024(EAEC), Tube 7:73A(ETEC), Tube 8: C19(EHEC), Tube 9: S04(STEC), Tube 10: C57(EIEC), Tube 11: C63(Nonpathogenic E. coli), and Tube 12:NC.**B)** Gel electrophoresis result; Lane M: DNA ladder(100plus), lane 1: 46A (EPEC), lane 2:C89, lane 3: C75, lane 4: C71, lane 5: CF10, lane 6: 024(EAEC), lane 7:73A(ETEC), lane 8: C19(EHEC), lane 9: S04(STEC), lane 10: C57(EIEC), lane 11: C63(Nonpathogenic E. coli), and Lane 12:NC.

Supplementary Table 1 :2x2 Contingency matrix table generated by MedCalc Version 23.2.1 Software.

| Test | Present | No | Absent | No | Total |
| --- | --- | --- | --- | --- | --- |
| Positive | TP | 10 | FP | 0 | 10 |
| Negative | FN | 0 | TN | 50 | 50 |
| Total | - | 10 |  | 50 | 60 |

Supplementary Table 2: Summary of serial dilution result, showing a comparative detection limit for both LAMP and PCR.

| Dilution Factor | DNA concentration | DNA concentration per reaction (2 µL template). | LAMP | PCR |
| --- | --- | --- | --- | --- |
| 10^-1^ | 25.1 ng/ µL | 50*10^1^ ng/reaction | **+** | **+** |
| 10^-2^ | 2.51ng/ µL | 5.50*10^0^ ng/reaction | **+** | **+** |
| 10^-3^ | 0.251ng/ µL | 5.50*10^-1^ ng/reaction | **+** | **+** |
| 10^-4^ | 0.0251ng/ µL | 5.50*10^-2^ ng/ reaction | **+** | **+** |
| 10^-5^ | 2.50 pg /µL | 5pg/reaction | **+** | **+** |
| 10^-6^ | 0. 251 pg /µL | 0.5 pg/reaction | **+** | **-** |
| 10^-7^ | 0.0251pg/µL | 0.05pg/reaction | **+** | **-** |
| 10^-8^ | 0.00251pg/µL | 0.005 pg/reaction | - | - |
| 10^-9^ | 0.000251pg/µL | 0.0005 pg/reaction | - | - |
| 10^-10^ | 0.0000251pg/µL | 0.00005 pg/reaction | - | - |
